# Swarm learning network for privacy-preserving and collaborative deep learning assisted diagnosis of fracture: a multi-center diagnostic study

**DOI:** 10.3389/fmed.2025.1534117

**Published:** 2025-07-03

**Authors:** Yi Xie, Xinmeng Wang, Huiwen Yang, Jiayao Zhang, Honglin Wang, Zineng Yan, Jiaming Yang, Zhiyuan Yan, Zhiwei Hao, Pengran Liu, Yijie Kuang, Zhewei Ye

**Affiliations:** ^1^Department of Orthopedics Surgery, Union Hospital, Tongji Medical College, Huazhong University of Science and Technology, Wuhan, China; ^2^Laboratory of Intelligent Medicine Research, Union Hospital, Tongji Medical College, Huazhong University of Science and Technology, Wuhan, China; ^3^Key Laboratory of Clinical Biochemistry Testing in Universities of Yunnan Province, School of Basic Medical Sciences, Dali University, Dali, China; ^4^Department of Otorhinolaryngology, Union Hospital, Tongji Medical College, Huazhong University of Science and Technology, Wuhan, China; ^5^Department of Orthopedics Surgery, Fujian Provincial Hospital, Fuzhou, China; ^6^School of Medicine, Wuhan University of Science and Technology, Wuhan, China; ^7^School of Artificial Intelligence and Automation, Huazhong University of Science and Technology, Wuhan, China

**Keywords:** blockchain, swarm learning, artificial intelligence, fracture, tomography, x-ray computed, deep learning, federated learning

## Abstract

**Background:**

While artificial intelligence (AI) has revolutionized medical diagnostics, conventional centralized AI models for medical image analysis raise critical concerns regarding data privacy and security. Swarm learning (SL), a decentralized machine learning framework, addresses these limitations by enabling collaborative model training through secure parameter aggregation while preserving data locality. However, no prior studies have specifically developed distributed learning models for fracture recognition due to challenges in multi-institutional data harmonization. We aimed to develop and validate a blockchain-based SL framework for privacy-preserving, multi-institutional fracture image analysis and compare its performance against centralized AI models and clinicians in real-world applications.

**Methods:**

We selected knee joint diseases in traumatic orthopedics as representatives to explore the AI imaging evaluation of fractures. The knee joint images were retrospectively obtained from patients diagnosed with knee injuries between December 2013 and July 2023 at 4 independent institutes hospitals in China. A total of 4,581 patients was included for retrospective study and establishment of the explainable and distributed SL model. An explainable object detection algorithm was proposed for the identification of fractures. Based on the architecture, a privacy-preserving SL system was established, and we further validated the performance of the model in external verification sets and clinical use. Finally, the SL system was appraised through a prospective cohort to aid 6 clinicians in the preoperative assessment of 112 patients with knee joint injuries.

**Results:**

The YOLOv8n-cls algorithm demonstrated superior performance in centralized experiments and was adapted for SL implementation. Our SL model achieved robust performance in both balanced (AUROC 0.991 ± 0.003, accuracy 0.960 ± 0.013) and unbalanced (AUROC 0.990 ± 0.005, accuracy 0.944 ± 0.021) datasets. External validation yielded an AUROC of 0.953 ± 0.016, matching centralized model performance while maintaining data privacy. Clinically, the SL system achieved 86.8% diagnostic accuracy and assisted treatment decisions in 91.5% of cases, outperforming junior clinicians and rivaling senior specialists in diagnostic efficiency.

**Conclusion:**

This study establishes blockchain-based SL as a secure, privacy-preserving paradigm for distributed AI training in medical imaging, with particular relevance for emergency orthopedic diagnostics. Our framework enables effective multi-center collaboration without compromising data security, addressing a critical need in modern healthcare AI.

**Clinical trial registration:**

[https://www.chictr.org.cn/showproj.html?proj=193847], identifier [ChiCTR2300070658].

## 1 Introduction

Bone fractures represent a growing public health concern, with increasing incidence rates paralleling the rapid development of modern society, particularly due to traffic accidents and industrial injuries. In emergency trauma settings, expeditious and precise diagnosis coupled with appropriate therapeutic intervention is paramount for optimal patient outcomes. Recent advancements in image processing and artificial intelligence (AI) have significantly contributed to bone fracture detection, offering robust methods for improving diagnostic accuracy and efficiency. Contemporary medical practice has witnessed the emergence of deep learning (DL) as a transformative paradigm in medical image analysis (1–4). Through sophisticated feature extraction and pattern recognition capabilities, DL methodologies have demonstrated remarkable efficacy in fracture identification, classification, lesion segmentation, and risk stratification (5–7). Extant literature has predominantly explored centralized computational architectures for fracture image analysis, including investigations of proximal femoral fractures, vertebral fractures, and clinical fracture prediction models (8–10). While these centralized approaches exhibit promising results, they are accompanied by substantial limitations and potential vulnerabilities that warrant critical examination (11–22). Such frameworks necessitate extensive, consolidated training datasets, which fundamentally impedes multi-institutional collaborative research due to restricted access to heterogeneous data repositories (13, 14). Additionally, conventional medical data acquisition methodologies are encumbered by ambiguities regarding data sovereignty, inter-organizational conflicts of interest, and departmental regulatory constraints. Patient privacy protection remains a paramount consideration in the development and implementation of DL-augmented diagnostic systems. Furthermore, the expansion of clinical feature models to encompass a broader spectrum of pathologies necessitates innovative technological solutions capable of seamlessly integrating multi-institutional datasets while maintaining data security and integrity.

## 2 Relevant literature

Over the past 5 years, decentralized machine learning paradigms have emerged as elegant solutions to the critical dual imperatives of leveraging advanced computational intelligence while maintaining stringent privacy safeguards (15). Within distributed architectural frameworks, individual nodes conduct autonomous deep learning (DL) model training using exclusively local datasets, eliminating the necessity for raw data transmission. This represents a fundamental departure from conventional centralized approaches, as decentralized learning protocols enable seamless multi-institutional collaboration (16). The DL training methodology involves periodic parameter exchange between participating nodes, facilitating collective model refinement while ensuring that each node’s data access remains strictly confined to its local repository.

In medical applications, blockchain technology provides a robust incentivization mechanism for institutional and individual participation in model development—an increasingly essential component of decentralized deep learning ecosystems (17). Blockchain’s inherent traceability functionality ensures equitable attribution and compensation for all contributing entities based on their specific inputs, including medical image annotation, dataset provision, and algorithmic innovation (18–22). There are some capabilities of blockchain technology in safeguarding sensitive healthcare data:

• Data encryption: blockchain leverages advanced cryptographic techniques such as public-key cryptography and hash functions to secure data. Each transaction on the blockchain is encrypted using a unique cryptographic key. The patient’s private data is encrypted before being added to the blockchain, ensuring that only authorized parties.
• Immutability: one of the defining features of blockchain is its immutability, meaning once data is written to the blockchain, it cannot be altered or deleted without the consensus of the network. This ensures the integrity of medical records, preventing unauthorized modifications, tampering, or data loss, which is critical in clinical settings where accurate, immutable records are paramount.
• Distributed ledger: the decentralized nature of blockchain means that data is stored across multiple nodes, rather than on a single centralized server. This distribution reduces the risk of single points of failure and enhances the security of medical records by ensuring redundancy. Furthermore, even if one node is compromised, the other nodes will continue to hold secure copies of the data, ensuring resilience against attacks.
• Access control: blockchain allows for granular access control mechanisms, utilizing smart contracts to define who can access specific data and under what conditions. For instance, healthcare providers can be granted access to patient data based on pre-defined, permissioned rules set within the blockchain. These smart contracts automate the verification process, ensuring that only authorized personnel can view or update clinical information, thereby maintaining both privacy and accountability.
• Auditability: blockchain’s transparent nature allows all transactions to be logged in an immutable ledger. This creates a comprehensive audit trail that can be accessed by authorized parties, ensuring full traceability of actions taken with respect to patient data. In clinical settings, this feature enhances compliance with regulations and allows for real-time monitoring of data access.
• Interoperability: blockchain facilitates secure data exchange between disparate healthcare systems by providing a unified and standardized platform for sharing patient records. Using interoperable blockchain networks, healthcare institutions can seamlessly and securely exchange data without compromising patient privacy.

The sophisticated approach empowers participants with comprehensive control over data authenticity and security while simultaneously benefiting from the enhanced diagnostic accuracy and performance metrics of the collaboratively developed model. Presently, swarm learning (SL) is considered to be an effective privacy-preserving method to train DL models through trusted and secure parameter sharing (23–27). The SL can be defined as an integrated training model that combines the advantages of AI, FL, and blockchain, so it is considered an advanced version of federated learning (FL). There are some advantages of SL over conventional AI and FL approaches (Supplementary Table S1). Different from traditional FL, the SL may provide a promising approach for optimizing clinical decisions through robust collaborative model training across different data sources (23, 27–30).

Rapid fracture diagnosis and patient transfer are critical for emergency care, particularly in resource-limited settings where primary healthcare facilities often lack capacity for radiographic fracture identification for occult fracture. This study presents the first implementation of SL for orthopedic fracture diagnosis, addressing critical limitations in resource-limited settings where conventional diagnostic capabilities are often unavailable. Our decentralized SL framework enables secure, multi-center collaboration while maintaining diagnostic accuracy comparable to centralized models, as demonstrated through systematic evaluation of TPF identification across distributed nodes with blockchain-secured aggregation. The clinically validated system combines automated fracture image analysis with privacy-preserving distributed learning and traceable data governance, achieving 86.8% diagnostic accuracy in prospective testing while overcoming key challenges in patient data privacy and cross-institutional collaboration. This work establishes a new paradigm for global orthopedic care by enabling secure knowledge sharing across healthcare tiers, maintaining diagnostic performance in variable resource settings, and providing an open-access implementation^1^ that bridges the gap between AI innovation and clinical deployment in trauma care (26).

## 3 Materials and methods

### 3.1 Medical image data collection

**FIGURE 1:**
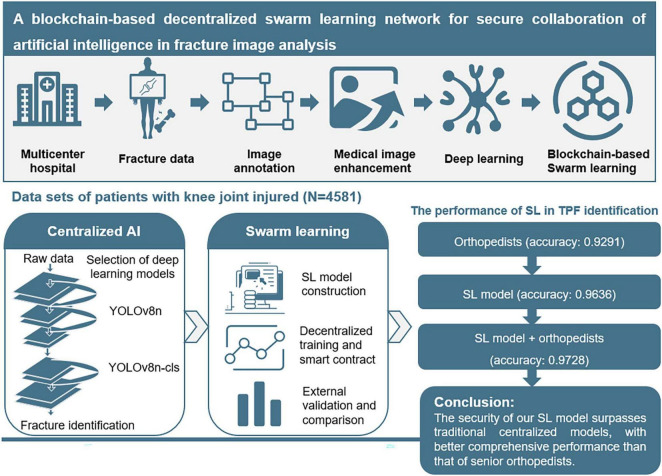
The schematic depiction of our study design process.

To train and validate local centralized algorithms, patient data were divided into training dataset (*n* = 3,027), internal validation dataset (*n* = 377), and testing dataset (*n* = 377), with a distribution ratio of approximately 8:1:1. Additionally, an external validation dataset, consisting of 800 knee X-ray images (400 with TPFs and 400 without), was used to compare the performance of the SL network, the centralized model, and radiologists. A detailed schematic of the study design and process is shown in Figure 1. The management of X-ray image data from four independent hospitals in China, the detailed data statistics can be found in Table 1. The inclusion and exclusion criteria of the patients are provided in Supplementary Table S2.

**TABLE 1:** Clinical classification and pathological features of patients from different hospital node of our blockchain-based network.

Statistical characteristics	WU	WF	HTCM	FP
Use in this study	Training	Internal validation	Testing	External validation
Cohort type	Population	Population	Population	Population
*N* Patients in cohort	3,027	377	377	800
Age (median)	45.94	43.68	38.89	N/A
Age (IQR)	12.08	10.12	13.66	N/A
Gender: male	2,367 (78.2%)	234 (62.1%)	189 (50.1%)	482 (60.3%)
Gender: female	660 (21.8%)	143 (37.9%)	188 (49.9%)	318 (39.7%)
Type I	195	25	24	92
Type II	320	42	40	64
Type III	169	18	20	50
Type IV	181	23	23	76
Type V	225	33	40	42
Type VI	200	20	13	20
Type K	95	11	12	48
Without TPF	1,642	205	205	400

WU, Wuhan Union Hospital; WF, Wuhan Fourth Hospital; HTCM, Hainan Traditional Chinese Medicine Hospital; FP, Fujian Provincial Hospital; N/A, not applicable; IQR, IQR, interquartile range; TPF, tibial plateau fracture.

Using medical processing software, we converted the X-ray images from DICOM format to high-definition JPG files. Fracture diagnoses were primarily based on knee joint medical images, supplemented by the patients’ medical history. Two chief physicians collaborated to establish the final diagnosis. To enhance object detection accuracy at the fracture site and refine the algorithm, image preprocessing and further optimization of image settings were performed. Supplementary Figure S1 provides a detailed illustration of the image labeling and identification process. The Labelme software package was used for manual labeling in this study. For the primary task, each input image was resized to 608 pixels along the longer dimension, while preserving the original aspect ratio by scaling the shorter side accordingly. This approach ensures effective processing during training while retaining key information. After automatically cropping the tibial plateau area based on the label box, all images were resized to 480 × 480 pixels to ensure model adaptability to different image sizes. Then, letterbox resizing was applied to the input images during detection to ensure maximal image preservation. The backbone consists of three modules: CBS, C2f, and SPPF. The CBS module is composed of 2D convolution, 2D BatchNorm, and the SiLU activation function. The number of blocks in the backbone was modified from 3-6-9-3 to 3-6-6-3. In the field of medical image augmentation, Generative Adversarial Networks (GANs) and conditional diffusion models have been demonstrated in the processing of image data and improving the radiographic image analysis. However, from an academic perspective, existing studies have not yet utilized diffusion models for medical data augmentation in identification of traumatic fracture. As a generative model, diffusion model has shown remarkable advantages in medical image generation and augmentation in recent years. By generating more fracture samples and enhancing the training data of the segmentation model, its performance can be effectively improved. To enhance dataset diversity and improve the model’s generalization ability, we used the advanced GAN-based and diffusion model for data augmentation.

### 3.2 Model training

#### 3.2.1 Establishment of centralized model

**FIGURE 2:**
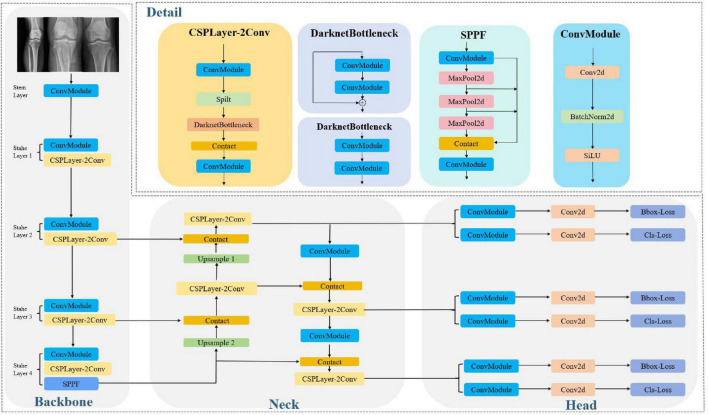
Structure of the YOLOv8 algorithm in TPF identification.

In the fracture detection task, we chose YOLOv8 to develop our model for recognizing the fracture areas, given its effective balance of detection accuracy and processing speed (31). Before being fed into the network, the original fracture images were standardized to a uniform size to ensure consistency. The specific architecture of YOLOv8 is shown in Figure 2. Additionally, letterboxing was applied during detection to ensure optimal image restoration by scaling the input image without distorting its aspect ratio. During training of the fracture recognition model, the first step involves extracting key features from the images in the training dataset. To enhance the richness of the dataset, advanced augmentation techniques such as image flipping, rotation, and cropping were used to increase diversity and expand the dataset’s scope. We employ a data augmentation strategy to expand the dataset (32). The features capture the essential patterns and characteristics needed for subsequent detection tasks. Once feature extraction is complete, the extracted features are passed to the neck module for further processing. The neck module utilizes the PAN-FPN structure to facilitate feature fusion, a key step that helps eliminate redundant detections and improves the accuracy of final fracture identification. After training, the performance of the YOLOv8 model was thoroughly evaluated using an independent test dataset. Evaluation metrics, including accuracy, sensitivity, and the false positive rate, were calculated to assess the model’s effectiveness in detecting fracture.

#### 3.2.2 Training of decentralized SL model

**FIGURE 3:**
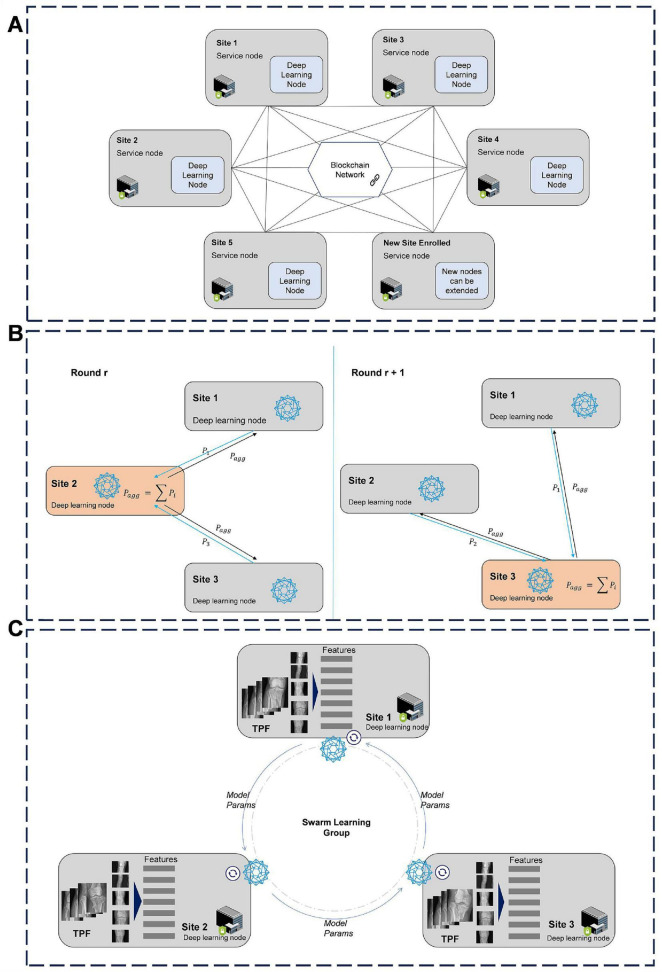
Distributed SL network architecture and training process of the fracture recognition model. **(A)** The proposed architecture of cooperative SL network in fracture image analysis. **(B)** Dynamic aggregation leader design of SL network. **(C)** Training process of TPF detection model based on swarm learning. SL, swarm learning; TPF, tibial plateau fracture.

The SL framework offers an alternative to centralized data aggregation from large patient cohorts, improving predictive accuracy and scalability while eliminating the need for central control over the final model (28, 29–33). We propose that blockchain-based decentralized SL solutions can address the limitations of current centralized learning approaches, meeting the growing demands of healthcare organizations and research units for decentralized data structures, as well as ensuring data privacy and compliance with security regulations (23, 34–36). To enable secure collaboration in training, we have developed an SL-capable AI cooperative network specifically for TPF detection. In the proposed decentralized training model, SL accommodates distributed data structures and computing devices, similar to FL. This approach ensures that data remains secure with its owner while enabling efficient model training. Additionally, SL ensures equal participation by allowing all network members to share rights and responsibilities. This is achieved through the dynamic assignment of an aggregation leader among all members, facilitated by a blockchain smart contract (37). As a result, all members alternately contribute to the calculation of shared parameters to aggregate the final model. Furthermore, the SL framework is compatible with a ring-all-reduce architecture, where the leader role can be topologically omitted, and each member performs part of the aggregation process concurrently (38–41). However, this architecture requires stable network connections and offers limited fault tolerance. To address these challenges, we have adopted a dynamic aggregation leader design within the distributed framework. As shown in Figure 3A, during each training round, parameters are updated based on local data and then synchronized with other deep learning nodes to update the shared global model (42, 43). Through smart contract governance, the model achieves high security and fault tolerance, effectively mitigating risks such as poisoning attacks by implementing threshold-based safeguards (39, 44–45).

At each node, a deep learning model is trained, and the parameters for TPF feature recognition are aggregated across the SL network to update the overall model. As shown in Sites 1, 2, and 3 of Figure 3B, the deep learning nodes receive instructions from service nodes and collaboratively execute the training process (18, 22, 34, 41, 44, 45). Each node in the network holds its own original healthcare data at the local site. We adapted the distributed SL model based on the previously trained YOLOv8 deep learning model to assess its effectiveness in terms of data security and performance. A comparative analysis was conducted between centralized, local, and SL models to evaluate the performance of the SL algorithms. The YOLOv8 code was specifically modified to ensure compatibility with our SL framework. Hyperparameters and configurations used in training were tailored for the experiment, while other settings followed the official best practices for YOLOv8.

In the application of SLmodels for traumatic orthopedics, blockchain technology can be utilized to deploy algorithms effectively. This approach integrates external expert knowledge and decision-making tools, significantly improving the accuracy and efficiency of diagnosing and treating traumatic orthopedic conditions. By leveraging a specialized disease database and a blockchain-based traceability system, an intelligent, closed-loop treatment system can be established. Regarding the security and privacy of patient information, the blockchain-based SL model training utilizes distributed data storage, point-to-point transmission, consensus mechanisms, and encryption algorithms. This approach ensures a decentralized structure for medical data sharing, safeguarding the storage, transmission, and traceability of patient data. The SL model is collaboratively trained by trauma orthopedics departments across multiple hospitals via a blockchain system and deployed on cloud servers. When AI-assisted diagnosis is required, hospital physicians submit consultation requests, with access controlled through smart contracts. Upon receiving authorization, the system directs the request to the SL model for decision-making support and returns the clinical report results to the physicians.

To practically apply the model in clinical settings, we designed an experiment with a specific focus on clinical applications in emergency care units. In this study, blockchain simulation nodes from three different hospitals in China were established. Independent servers were used to configure deep learning tasks through the platform, with key elements such as participants, datasets, deep learning algorithms, and initial parameters being defined. As shown in Figure 3c, privacy computing networks were employed to distribute participating member nodes and upload the basic information of the training task to the SL, thereby creating the training task. Once the participants obtained the task information, they could invoke the interface services of the DL module on the computing engines of each node. The local training processes were launched based on the selected model, aggregation algorithm, and encryption scheme. The blockchain node was responsible for synchronizing the model’s calculation status. Meanwhile, a smart contract deployed on the consortium blockchain randomly selected one participant as the model aggregator for the current training round and disseminated this information to all member nodes. The model aggregator then initiated the aggregation process and made the aggregated model available to all participating nodes for further communication and scheduling. Member nodes shared their locally updated parameters with the model aggregator through the privacy computing network, utilizing a single encoding aggregation algorithm. Throughout the deep learning model training process, task status and training metrics were synchronously and in real time updated to the blockchain. When training was completed, participating nodes shared the trained model parameters. Throughout the entire training process, the members’ data resources remained within their respective domains, minimizing the risk of data leakage. The jointly trained model demonstrated higher accuracy compared to models trained with a single data source.

The blockchain-based SL model parameter updating and aggregation process is facilitated through the use of smart contracts. These contracts receive model parameters from the distributed ledger, aggregate them, and transmit the updated parameters back to the corresponding client-side ledgers. In this study, the distributed modeling process involves three trauma orthopedic departments across different hospitals. Each hospital trains the model using its local fracture data and, at the conclusion of each round, sends the updated model parameters—comprising weights and biases—back to the server for aggregation. Once aggregated, these parameters are sent back to the respective hospital nodes, where the models are updated with the newly aggregated parameters. This iterative process continues until a predefined number of rounds is completed.

### 3.3 Evaluation of model

**FIGURE 4:**
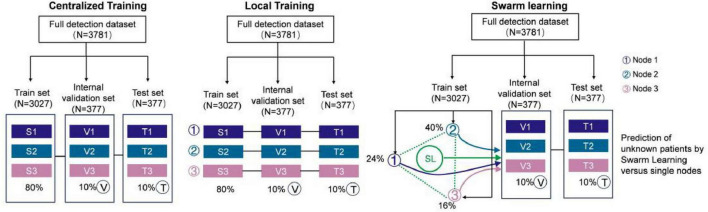
Data setup for comparative experiment and data division of different equilibrium degrees of each node in SL. Data setup in centralized AI, local training, and swarm learning.

To reduce statistical bias in data partitioning, the dataset was divided into three subsets for training, validation, and testing, with a ratio of 8:1:1. In the SL node sets, to better reflect real-world trauma center databases, the training set is further split into three non-overlapping subsets with a ratio of 5:3:2, accounting for the scale differences among medical organizations. The test sets from each subset are combined to form the global test set. The data training process across various modes of the SL model is visualized in Figure 4. In centralized training scenarios, the training set combines data from all participants. The models are trained for 100 epochs, and the best checkpoint is selected using the global validation set. In local training scenarios, models are trained on local training sets for 100 epochs, with the best model selected using the global evaluation set. In SL cases, the collaborative model is trained for 100 rounds, with each node running one epoch on its local training set per round. The best checkpoint is selected during training using the global validation set. After training, the final models from all scenarios are evaluated on the global test set to compare their performance. Additionally, external validation (*n* = 800) from Fujian Provincial Hospital was used to assess the stability of the SL model in clinical settings.

We thoroughly evaluated the impact of imbalanced data on model performance and accuracy in more realistically and reasonably clinical data. Typically, SL requires independent and identically distributed (IID) data across sites to achieve performance comparable to centralized models (30, 45). However, real-world datasets often exhibit non-IID characteristics due to variations in disease presentations, imaging protocols, or patient demographics. These differences can degrade model performance. Previous studies have shown that uneven data distributions can lead to a decline in the accuracy of distributed learning models. When local data distributions among nodes differ, it may compromise the fairness and robustness of the trained models. To ensure the effectiveness and robustness of our SL models, we performed tests in both equilibrium and non-equilibrium states. In the balanced data distribution, training sets for each node were randomly sampled, with controls almost evenly distributed across all three nodes (22, 28–30, 34, 45–47). In the unbalanced data distribution, training sets for different nodes were non-IID, representing extreme cases that reflect challenges may faced by real-world distributed learning systems. The detailed data distributions for both scenarios are shown in Table 2.

**TABLE 2:** The balanced and unbalanced data distribution in the SL model training.

Trauma types		Node 1 (*n* = 1,513)	Node 2 (*n* = 908)	Node 3 (*n* = 605)
**The balanced data distribution**				
TPF	Schatzker I (class A)	94	57	44
	Schatzker II and III (class B)	249	133	107
	Schatzker IV (class C)	83	61	37
	Schatzker V and VI (class D)	217	135	73
	Intercondylar ridge fracture (class K)	49	29	16
Without TPF		821	493	328
**The unbalanced data distribution**				
TPF	Schatzker I (class A)	–	–	195
	Schatzker II and III (class B)	–	489	–
	Schatzker IV (class C)	181	–	–
	Schatzker V and VI (class D)	425	–	–
	Intercondylar ridge fracture (class K)	–	94	–
Without TPF		907	325	410

To investigate whether the use of a SL model can enhance diagnostic performance while preserving privacy, a benchmark study was designed to compare the proposed system with a baseline model based on centralized learning. The baseline model, previously developed by our team, employs deep learning with the original RetinaNet architecture (48–51). For this study, we retrained the RetinaNet base model and used YOLOv8-cls for identification and classification tasks. Training and validation followed the same procedures as those for the SL model, with Regions of Interest (ROI) used as the input for both models. All image preprocessing techniques and hyperparameter settings for the SL network remained consistent with those outlined in our previous study. To validate our SL-based methodology for predicting fractures from X-ray images, we conducted a clinically relevant prediction task comparing our system’s performance with that of orthopedists. Six orthopedists participated in the study, including two senior orthopedists with 12 years of clinical experience, two attending orthopedists with 6 years of experience, and two orthopedic residents with 2 years of experience. A subset of 200 cases from an external validation set was randomly selected to compare the diagnostic performance between the centralized AI model, the SL system, and the human doctors. Each expert was asked to make a comprehensive judgment on the observed X-rays, including determining whether the knee was fractured, identifying the fracture site, and classifying the type of fracture. None of the test cases had been previously seen by any of the experts. The cases were anonymized, shuffled, and stored on a password-protected computer, along with a spreadsheet documenting each expert’s diagnosis.

The validated model is integrated into the interface with special access rights in the hospital imaging system, which can automatically evaluate the TPF in an end-to-end manner, from the original X-ray image input to the generation of interpretable diagnosis. In order to evaluate the feasibility of assisting orthopedic doctors in a clinical environment, the system was used in a prospective cohort of knee trauma patients who visited the hospital in a single arm observational study. The model provide the predicted results of fracture location and classification, together with routine evaluation, to two senior orthopedic doctors, who have the right to decide the treatment and operation in various ways. We also conducted a survey of surgeons on the use of models to generate information in the decision-making process of these cases. Model prediction is used to analyze the choice of surgical approach, and measure the performance of the model according to the radiographic results. The recovery of TPF was evaluated by comparing the range of motion and anatomical reduction of knee joint after conservative treatment or 6 months after operation (52).

### 3.4 Statistical analysis

To evaluate the effectiveness of our deep learning model, we established various probability thresholds and assessed performance across different fracture classifications. Key metrics, including accuracy, sensitivity, F1 score, and AUC, were used to evaluate the model’s performance in fracture typing. Precision-recall (PR) curves and average precision (AP) scores were employed to assess the efficacy of the multi-class classification algorithm. Each of these metrics plays a crucial role in fine-tuning the model to ensure its effectiveness in diverse applications. After 5 iterations of cross-validation or external validation, we averaged the training results and reported them as mean (SD). For assessing internal consistency, we used the Cohen kappa coefficient. Performance differences between models were evaluated using a two-tailed paired *t*-test, while one-way analysis of variance (ANOVA) was used to compare the proposed model against human experts in clinical application. The alpha level is set when conducting statistical analysis using Python 3.10.9. (48).

## 4 Experimental results

This section details the experimental outcomes of our study, which evaluated the performance of the two customized models—centralized YOLOv8 algorithm and SL network designed for automatic bone fracture detection from X-ray images. The training parameters of centralized model was shown in Table 3. The aggregation algorithm settings of SL were shown in Table 4.

**TABLE 3:** Centralized learning and training parameters.

Parameter category	Parameter	Configuration
Model architecture	Architecture	YOLOv8n
	Feature extractor	CSP-Darknet backbone
	Detection head	Multiple anchors with objectness prediction
	Input resolution	608 × 608 pixels (letterboxing applied)
Optimization	Base optimizer	SGD with momentum
	Momentum coefficient	0.937
	Weight decay	0.0005
	Initial learning rate	0.01
	Learning rate schedule	Cosine decay with warm-up (5 epochs)
	Minimum learning rate	1 × 10^–5^
	Batch size	16 per node
	Training epochs	100
Loss function	Object detection	CIoU loss (α = 0.5)
	Classification	Binary cross-entropy
	Box regression	Complete IoU loss
	Objectness	Focal loss (γ = 1.5)

**TABLE 4:** SL model training and parameters setting.

SL	Aggregation algorithm	Weighted averaging (Fed-Avg)
	Aggregation frequency	Every 10 local batches
	Local update steps	5 per round
	Minimum node participation	3 nodes
	Parameter encryption	Diffie-Hellman key exchange protocol
	Blockchain consensus	Practical Byzantine Fault Tolerance
Data processing	Augmentation techniques	Random affine (± 15°), horizontal flip, HSV shifts (± 10%)
	Mosaic augmentation	Applied with 4-image composition
	Mixup probability	0.15
	Normalization	Mean subtraction and scaling (μ = 0.485, 0.456, 0.406; σ = 0.229, 0.224, 0.225)
Evaluation	Confidence threshold	0.25
	NMS IoU threshold	0.45
	Validation frequency	Every epoch

### 4.1 Environment setup

The experiments and SL simulations in this paper were carried out using three AMAX GPU servers, each equipped with two Intel(R) Xeon(R) Gold 6226R CPUs (2.90GHz), 24TB HDD, 256GB RAM, and four NVIDIA Tesla V100S GPUs. High-speed kMbps interconnections link the servers.

### 4.2 YOLOv8n’s performance in identification of TPF

**FIGURE 5:**
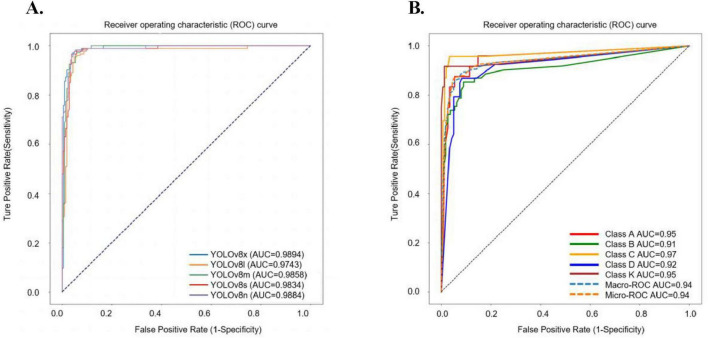
The ROC curve results of five YOLOv8 sub-models in fracture detection and the ROC curves for different classifications using YOLOv8n-cls. **(A)** In the task of detection of TPF, the AUC value for YOLOv8n is 0.9884, for YOLOv8s is 0.9834, for YOLOv8m is 0.9858, for YOLOv8l is 0.9743, and for YOLOv8x is 0.9894. **(B)** In our centralized model, the AUC values of each type in the ROC curve: class A is 0.95, class B is 0.91, class C is 0.97, class D is 0.92, class K is 0.95.

**FIGURE 6:**
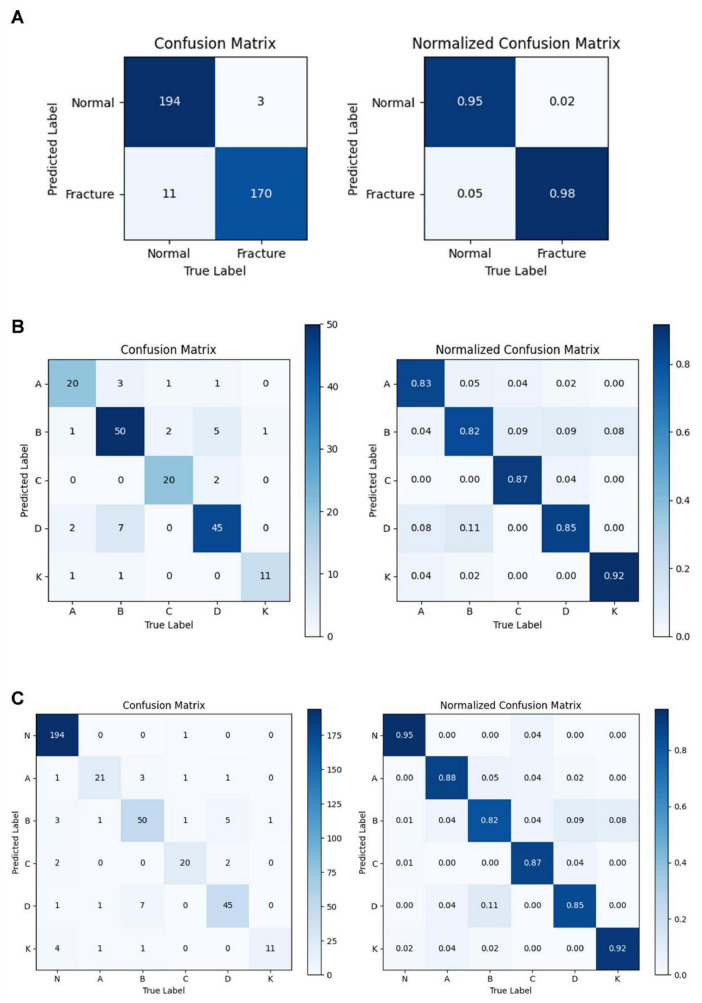
The predict results of fracture images analysis. **(A)** The confusion matrix and the normalized confusion matrix of YOLOv8n in TPF detection. **(B)** The confusion matrix and the normalized confusion matrix of YOLOv8n-cls for TPF single classification task. **(C)** The confusion matrix and the normalized confusion matrix of YOLOv8n-cls for TPF identification and classification task.

This initial experiment centralized fracture imaging data on a server and trained the AI model on a multi-level combined dataset to assess its efficiency. Among the models tested, YOLOv8n demonstrated the highest Youden index, with a threshold score of 0.5493. The intersection over union (IoU) for YOLOv8n was calculated at 0.8845, highlighting a close alignment between the model’s generated detection boxes and the doctor-labeled boxes, fully encompassing the tibial plateau regions. Detailed results for all models are provided in Supplementary Table S3 and Figure 5. In testing, YOLOv8n excelled in detecting TPF, achieving an accuracy of 0.9632, sensitivity of 0.9884, and specificity of 0.9366. The confusion matrix for TPF detection is presented in Figure 6.

### 4.3 Comprehensive analysis of fractures using decentralized models

As is seen in Table 5, in the distributed balanced data set, the accuracy of the three nodes was 0.939 (SD 0.032), 0.944 (SD 0.016), and 0.929 (SD 0.021), while the global model achieved an accuracy of 0.960 (SD 0.013). In the unbalanced data set, the accuracy of the SL model at the three nodes was 0.934 (SD 0.042), 0.931 (SD 0.053), and 0.926 (SD 0.042), with the global model achieving an accuracy of 0.944 (SD 0.021). It is evident that the overall performance of the SL model in balanced data sets surpasses that in unbalanced data sets. Further analysis of additional metrics such as recall, specificity, precision, F1-score, and AUC revealed that each node’s performance in the unbalanced group was inferior to that in the balanced group, confirming that model performance declines with imbalanced medical data. Compared to the centralized model, individual nodes (node 1, node 2, node 3) performed significantly worse in both balanced and unbalanced data distributions (*P* < 0.001). However, after integrating blockchain and SL fusion, the performance difference between the global model and the centralized training model became negligible. In the balanced data set, the global model demonstrated accuracy of 0.960 (SD 0.013), recall of 0.935 (SD 0.022), specificity of 0.937 (SD 0.017), precision of 0.961 (SD 0.018), F1-score of 0.925 (SD 0.021), and AUC of 0.991 (SD 0.003), showing no significant difference from the centralized model (*P* = 0.33). Similarly, in the unbalanced data set, the global model maintained comparable performance metrics: accuracy of 0.960 (SD 0.013), recall of 0.935 (SD 0.022), specificity of 0.937 (SD 0.017), precision of 0.961 (SD 0.018), F1-score of 0.925 (SD 0.021), and AUC of 0.991 (SD 0.003), with no significant difference observed compared to the centralized model (*P* = 0.26). These results indicate that our model can achieve diagnostic performance on par with centralized models under both balanced and imbalanced conditions, while preserving data privacy and security.

**TABLE 5:** Performance of the baseline centralized AI and the proposed SL model in TPF identification.

Data distribution		Accuracy mean (SD)	Recall mean (SD)	Specificity mean (SD)	Precision mean (SD)	F1-score mean (SD)	AUROC, mean (SD)	*P*-value
Centralized model		0.963 (0.016)	0.987 (0.032)	0.937 (0.047)	0.946 (0.039)	0.925 (0.012)	0.990 (0.001)	N/A
Balanced data	Node 1	0.939 (0.032)	0.949 (0.051)	0.912 (0.054)	0.947 (0.044)	0.883 (0.028)	0.977 (0.012)	< 0.001
	Node 2	0.944 (0.016)	0.951 (0.043)	0.942 (0.049)	0.952 (0.042)	0.889 (0.015)	0.974 (0.008)	< 0.001
	Node 3	0.929 (0.021)	0.923 (0.036)	0.912 (0.046)	0.927 (0.038)	0.860 (0.018)	0.979 (0.005)	< 0.001
	Trained on all (SL)	0.960 (0.013)	0.935 (0.022)	0.937 (0.017)	0.961 (0.018)	0.925 (0.021)	0.991 (0.003)	0.33
Unbalanced data	Node 1	0.934 (0.042)	0.892 (0.058)	0.917 (0.049)	0.901 (0.047)	0.871 (0.024)	0.979 (0.011)	< 0.001
	Node 2	0.931 (0.053)	0.918 (0.052)	0.893 (0.041)	0.934 (0.035)	0.870 (0.037)	0.948 (0.013)	< 0.001
	Node 3	0.926 (0.042)	0.906 (0.038)	0.907 (0.045)	0.911 (0.042)	0.855 (0.029)	0.962 (0.009)	< 0.001
	Trained on all (SL)	0.944 (0.021)	0.933 (0.036)	0.922 (0.043)	0.948 (0.039)	0.893 (0.037)	0.990 (0.005)	0.26

AUROC, areas under the receiver operating characteristic curve; N/A, not applicable.

**FIGURE 7:**
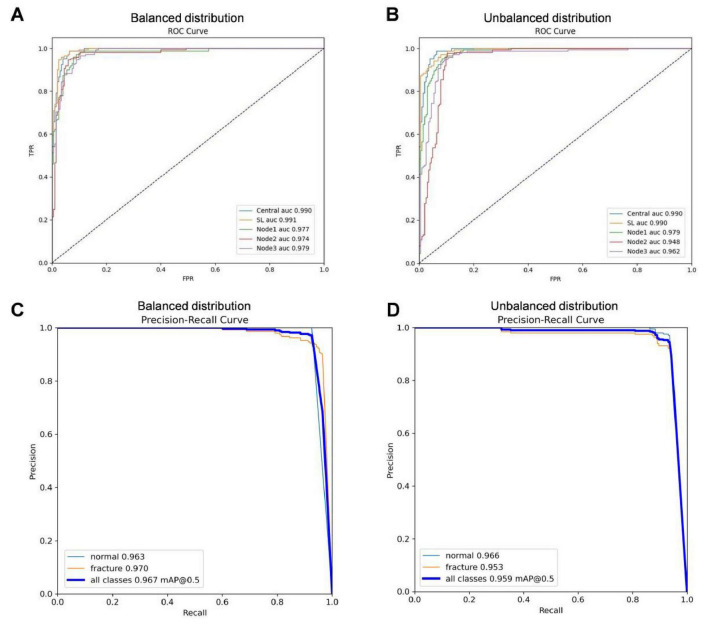
The experimental result of centralized AI and SL in balanced and unbalanced data distribution scenario. **(A)** ROC curve of SL in balanced data distribution. **(B)** ROC curve of SL in unbalanced data distribution. **(C)** PR-curve of SL in balanced data distribution. **(D)** PR-curve of SL in unbalanced data distribution (FPR: False positive rate).

Statistical analysis revealed no significant difference in performance between the SL model trained on all data and the centralized model for both datasets, indicating the stable computational efficiency of the SL approach. As shown in Figure 7, In the balanced data set, the AUC values of SL was 0.991 (SD 0.003), while in the non-balanced data set, the AUC values of SL was 0.990 (SD 0.003), and there was no statistical difference between them (*P* = 0.2668). In the PR curve, the mAp50 value of the SL model was 0.9590 for the unbalanced data set and 0.9665 for the balanced data set. These values are close to the mAp50 value of the centralized AI model (0.9678), and the efficiencies of both are comparable. This demonstrates the SL model’s excellent balance between predictability and efficiency. Overall, these results highlight the SL model’s remarkable capability for TPF recognition.

### 4.4 Interpretability of the swarm-trained model in external validation sets

**FIGURE 8:**
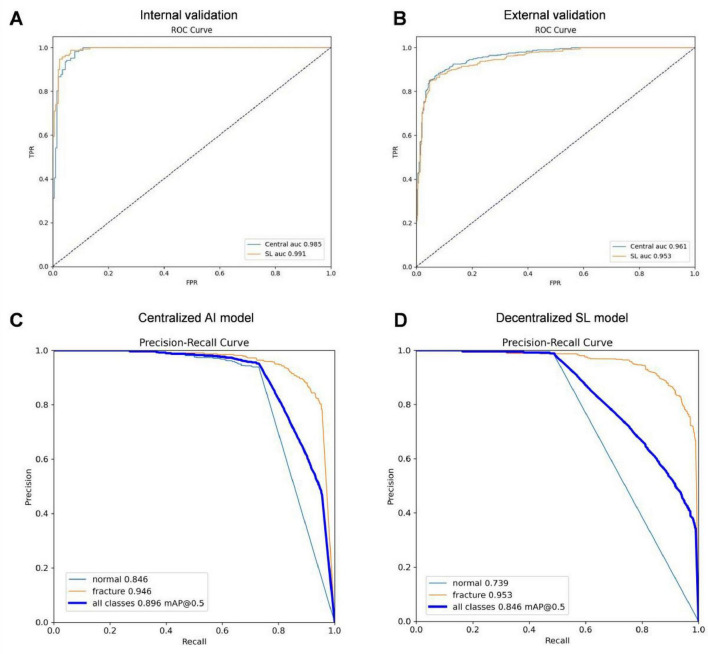
The performance of the centralized model and SL in the internal and external validation sets. **(A)** ROC curve comparison of centralized AI model and SL model in internal validation set. **(B)** ROC curve comparison of centralized AI model and SL model in external validation set. **(C)** PR-curve of centralized SL model in external validation set. **(D)** PR-curve of SL model in external validation set.

To further assess the experimental performance of the SL model on external datasets, we selected 800 patients from real-world clinical scenarios, beyond the retrospective cohort used in this study, for external validation. Table 6 presents the evaluation metrics and TPF detection performance of both the SL and centralized models. As shown in Figure 8, on the internal validation dataset, the SL model achieved an AUC of 0.991, surpassing the centralized AI model, which had an AUC of 0.985. On the external validation set, the ROC curve of SL showed an AUC of 0.953 (SD 0.016), while the AUC of the centralized model was 0.961 (SD 0.016), the difference was not statistically significant (*P* > 0.05). The use of SL enhances data privacy and facilitates collaboration across different agencies while maintaining secure AI model training. for the external dataset, the centralized model achieved a mean average precision (mAP) of 0.896, slightly higher than the SL model’s mAP of 0.846. However, when specifically evaluating the detection of TPF fractures (depicted in the orange PR curve), the centralized AI model achieved an AUC of 0.946, while the SL model achieved a slightly higher AUC of 0.953. These results indicate that both models exhibit comparable high accuracy in detecting TPF fractures, with AUC values approaching 0.950. This suggests that both the centralized and SL models offer clinically relevant performance in fracture detection, demonstrating their practical significance in real-world applications.

**TABLE 6:** TPF detection performance in the external data set.

	Accuracy mean (SD)	Recall mean (SD)	Specificity mean (SD)	Precision mean (SD)	F1-score mean (SD)	mAp50 mean (SD)	AUROC mean (SD)	*P*-value
SL network	0.9000 (0.012)	0.740 (0.014)	0.923 (0.025)	0.821 (0.027)	0.778 (0.033)	0.846 (0.028)	0.953 (0.016)	< 0.003
Centralized model	0.9038 (0.009)	0.8425 (0.016)	0.9225 (0.023)	0.8600 (0.021)	0.8512 (0.026)	0.8960 (0.024)	0.961 (0.016)	<0.001

### 4.5 Clinical implementation assessment

Timestamps were recorded during training execution and analyzed to compare time consumption between centralized training and SL. During the study, the SL training process took 5073.86 s, with an additional 2.10 s spent on encryption calculations, which was longer compared to the 2318.3224 s required for local centralized training. Our investigation revealed that the SL model matched the level of centralization in the aspect of accuracy, precision, sensitivity, specificity, and F1-score. The relevant metrics are depicted in Table 7. The decentralized SL model achieved a commendable balance between performance and privacy-preserving, and demonstrated superior diagnostic capabilities over attending orthopedists. The accuracy of SL network was 0.9636 [95% (0.9388, 0.9762)], the mean accuracy of orthopedists was 0.9291 (0.9002, 0.9482). And the SL model demonstrated a precision of 0.9526 [95% CI (0.9122, 0.9648)], while the precision of orthopedic attending physicians were 0.9057 [95% CI (0.8661, 0.9413)], the differences between these values were statistically significant (*P* < 0.05). Additionally, the sensitivity of the SL model was higher than that of orthopedic attending physicians (0.9837 vs. 0.9523). Regarding the misdiagnosis rate, the SL model exhibited a lower rate compared to orthopedic attending physicians (0.0163 vs. 0.0477). This study further analyzed the average diagnostic efficiency of five orthopedic physicians in traumatic TPF cases when assisted by distributed SL network. The findings indicated that, compared to orthopedic physicians without SL assistance, those with distributed SL support achieved an increased diagnostic accuracy of 0.9728 [95% CI (0.9602, 0.9882)], with precision rising to 0.9548 [95% CI (0.9433, 0.9575)] and sensitivity improving to 0.9848 [95% CI (0.9723, 0.9975)]. Both the YOLOv8n model and the distributed SLmodel exhibit considerable potential for identifying traumatic new TPF. In emergency scenarios, initial experimental results revealed that their diagnostic efficacy surpassed that of attending orthopedic physicians (*P* < 0.05). In addition, the time taken by the SL model (5.06 ± 0.02 min) was significantly less than that of the orthopedic attending physicians (25.45 ± 1.92 min) (*P* < 0.05). With the assistance of the SL model, the diagnostic efficiency of orthopedic physicians was significantly enhanced, and the average diagnostic time was reduced to 15.58 ± 2.62 min. This indicates that collaborative SL models could not only be securely cooperative but also substantially enhance diagnostic efficiency for orthopedic surgeons without compromising accuracy. Although the computational time of the algorithm will fluctuate within a certain range and be determined by factors such as network status, machine computing capacity, and current load, SL-capable AI can be more efficient and tolerant than centralized AI and human doctors.

**TABLE 7:** Comparison of diagnostic performance between orthopedic physicians and SL model.

Evaluation index	SL model	Orthopedists	Orthopedists + SL	*P-*value
Accuracy (95% CI)	0.9636 (0.9388, 0.9762)	0.9291 (0.9002, 0.9482)	0.9728 (0.9602, 0.9882)	0.004
Precision (95% CI)	0.9526 (0.9122, 0.9648)	0.9057 (0.8661, 0.9413)	0.9548 (0.9433, 0.9575)	0.004
Sensitivity (95% CI)	0.9837 (0.9510, 0.9918)	0.9523 (0.9159, 0.9755)	0.9848 (0.9723, 0.9975)	0.004
Time consumption (x ± s min)	5.06 ± 0.02	25.45 ± 1.92	15.58 ± 2.62	0.004

With the assistance of SL, orthopedic surgeons exhibited a significant improvement in the accuracy of TPF identification compared to the gold standard of actual fracture conditions. As shown in Table 8, in the internal validation set, the Kappa value increased from 0.838 (without SL assistance) to 0.910 (with SL assistance). Similarly, in the external validation set, the Kappa value increased from 0.769 (without SL assistance) to 0.840 (with SL assistance).

**TABLE 8:** Comparison of diagnostic consistency between orthopedic physicians and the gold standard in different datasets before and after using the SL model.

Dataset	Diagnosis result	Compare with the gold standard	
		k_1_*	k_2_*
Internal validation dataset	TPF	0.838	0.910
	Without TPF	0.859	0.930
External validation dataset	TPF	0.769	0.840
	Without TPF	0.851	0.880

*k1 represents the Kappa value for the comparison between the orthopedic physician’s diagnosis of X-ray images and the gold standard without SL assistance, while k2 represents the Kappa value for the same comparison with SL assistance.

**FIGURE 9:**
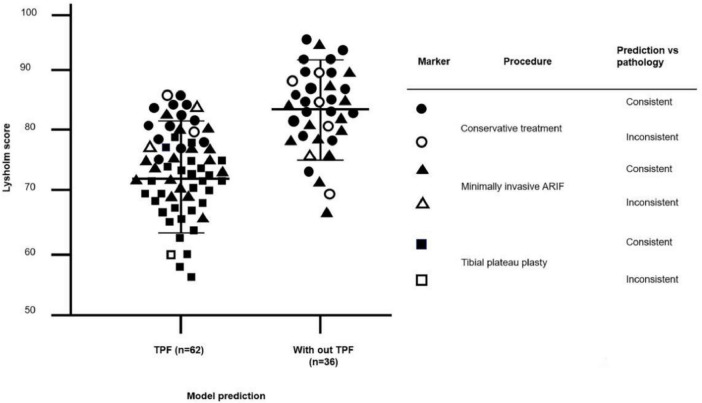
Functional recovery of the knee joint after treatment was evaluated in a total of 98 patients with available data. Scores were classified based on model predictions. Predictions consistent with pathological results were represented by closed symbols, while open symbols indicated inconsistencies. The circle denoted the conservative treatment group, the triangle represented the minimally invasive treatment group, and the square represented tibial plateau plasty. The error bar indicated ± 1 SD from the mean. TPF, tibial plateau fracture. ARIF, arthroscopic assisted reduction and internal fixation.

In the context of selecting treatment methods for knee joints, SL also offers support for intelligent decision-making processes. The automatic evaluation system, incorporating the validated SL network, was evaluated for its viability in aiding preoperative assessment in real-world settings involving 112 patients with knee joint injuries from Wuhan Union Hospital and Fujian Provincial Hospital (mean age 40.5, SD 13.2 years, 57.1% male). This system achieved an overall accuracy of 0.868 in distinguishing between TPF and without TPF cases. Seventy-six knees were identified as TPF by the model, all of which received arthroscopic assisted treatment of TPF. The patient’s data is distributed across a blockchain platform within the servers of the respective hospital, and the final training model parameters are utilized for fracture diagnosis through model scheduling. In the cases reported by clinicians, 91.5% (102/112) of the model predictions were consistent with their initial judgments or helped them make decisions. Compared with before treatment, 87.5% (98/112) patients achieved maximum recovery of knee function. Fracture-to-surgery interval shortened from 6.2 ± 1.8 days to 3.1 ± 0.9 days. Compartment syndrome incidence decreased by 42% (*P* = 0.03) due to earlier fasciotomy decisions, and the ICU admission rate reduced from 28% to 11% (*P* = 0.047). For the type of knee injury identified by the model, knee function was evaluated using Lysholm score after treatment in terms of lameness, swelling, behavioral support, and stability (52, 53). As is shown in Figure 9, Both groups of patients showed normal recovery, with a mean knee function score of 72.5 (SD 10.2) and 83.6 (SD 8.5).

## 5 Discussion and comparative analysis

This study substantiates the rationality of the application of blockchain based SL network in the collaborative analysis of orthopedic medical images and the practical worth of evading privacy disclosure during the model training. Compared to the centralized model, the SL network we proposed prevent illegal participants or potentially dangerous individuals, and provides a democratic approach to address the leader problem in model training. In this decentralized model, partners of each node communicate and work on an equal level (23–25). The different nodes jointly train medical models and share research results without compromising patient privacy or maintaining normal information governance, which is a new collaborative model required for the development of current medical models.

Nowadays, AI has demonstrated significant potential in the field of medical image recognition, but it has encountered a critical bottleneck and entered a stage of stagnation due to data acquisition posing as the primary obstacle to the development of large-scale models (6, 8, 10, 13, 15, 23, 34). As the medical community’s need to enhance data privacy and security continues to increase, distributed models will become the preferred option for management and analyzing a variety of large clinical and biological databases. To train medical AI models with high accuracy and strong generalization ability, relevant research institutions need to collaborate without compromising patient privacy. In fact, AI applications in the field of orthopedics had already begun to emerge (2, 6, 7). However, there is several frameworks have been developed to fortify the privacy and security in medical AI training, especially in department of orthopedics. In our study, we constructed orthopedic datasets from multiple centers across hospitals in China and proposed a blockchain-based SL model for distributed deep learning collaboration in real-world clinical settings. The model’s superior performance was validated in the recognition of medical images of traumatic fractures.

This study demonstrated the distributed model training approach and the collaborative use of intermediate reasoning results to enable comprehensive fracture analysis. The framework incorporates key data security measures: (1) Data isolation: Intermediate reasoning findings are isolated from the original data, ensuring that remote hospitals receive only analysis results without access to the underlying data sources or diagnostic processes. (2) High-level evidence: Clinical findings used for cross-hospital collaboration are derived from high-level evidence generated by AI-assisted expert systems. (3) Data encryption: Medical images are encrypted during synchronization, restricting access to authorized hospitals and safeguarding against cyberattacks. Furthermore, clinicians conduct patient inquiries to gather medical histories, and concerns regarding information exposure are mitigated through patient authorization for medical record usage and robust security protocols. While the current application study focuses on TPF identification, the proposed system is adaptable to other clinical domains. For instance, it can leverage multicenter data for precise mortality risk prediction, rehabilitation outcome assessment, and risk warnings for general practitioners. Implementing more implicit collaboration methods will further promote adoption in data-sensitive environments, ensuring both clinical utility and patient privacy.

As a multi-center study at the intersection of computer and medical fields, we constructed and validated the SL model for detecting TPF using X-ray images of 4,581 participants. In centralized local training, the training of DL models involves many prerequisites in terms of data preparation, with many pain points being addressed through dispersed medical datasets. Our proposed computational SL network utilizes the large dataset collected by our team to demonstrate the enormous potential of this novel collaborative approach for improving the clinical performance of doctors. In our prior work, we constructed different orthopedic AI diagnostic models based on multimodal data for trauma and fracture images, which including the prediction of lumbar spondylolisthesis fractures, wrist fractures, classification of femoral neck fractures, and the lung cancer bone metastasis (51, 52, 54–56). The application of AI in internet-based medical research and clinical settings has raised significant concerns, particularly related to the collection of large datasets and associated ethical issues. The international and multi-center collaboration supporting the proposed SL model stands to benefit greatly from advancements in data standardization for fracture classification and image analysis. By utilizing a secure and reliable training approach, engineers and clinicians can develop effective AI models without direct access to raw datasets, leveraging blockchain platforms to ensure data privacy. The SL framework presented in this study avoids dependence on a single model, minimizing the risk of bias and overfitting while safeguarding patient privacy.

Building upon our previous research, we have developed an advanced collaborative framework for AI model training in medical imaging (51, 52, 54–56). This framework utilizes a decentralized architecture, eliminating the dependency on a central coordination hub and enhancing flexibility for deployment across multiple healthcare institutions. By integrating blockchain technology with a distributed component, the framework facilitates collaborative training and AI-assisted diagnosis of medical images. Specifically, the local knowledge module processes and reasons with local data, while the distributed component manages the coordination of multicenter training processes. Our system has demonstrated the ability to identify previously overlooked fractures in advance, offering significant clinical benefits by alerting clinicians to fracture risks that might otherwise be missed. The results from our application study indicate that the proposed system can: (1) prevent delayed or missed diagnoses, (2) reduce unnecessary diagnostic tests, and (3) provide actionable diagnostic suggestions to support clinical decision-making. In the application study, 112 patients with knee joint injuries were evaluated. Clinician assessments revealed that 91.5% (102/112) of the model’s predictions aligned with their initial judgments, and 87.5% (98/112) of the evaluated patients exhibited positive symptoms. These findings suggest that a substantial proportion of patients could benefit from our system for timely TPF diagnosis, enabling prompt treatment and improving overall healthcare quality. Additionally, the system addresses the challenge of information gaps that often arise during patient transfers between hospitals. By facilitating secure information transmission, it provides risk alerts and clinical decision support during the initial post-transfer consultation, substantially decreases the incidence of fracture complications and streamlines the workflow of trauma orthopedic emergencies by intervening at an early stage in patients suspected of having TPF.

From the perspective of practical implications, the adoption of distributed models has enhanced the efficiency of data aggregation, ensured the security of traditional centralized AI models, and improved the diagnostic efficiency of medical professionals in clinical settings. This system enables dynamic aggregation of training parameters for each node, without the need for isolated agent nodes. Through this approach, we are able to monitor and ensure the realism and accuracy of overall training, while also providing timely feedback on the latest training outcomes. The proposed solution allows organizations to train deep learning models using others’ datasets without transferring their own datasets to an off-site location. In this study, the SL model outperformed junior clinicians and demonstrated equivalent performance to senior experts and centralized AI model in identifying TPF based on X-images. Computed tomography (CT) remains the reference standard for fracture classification, yet radiography persists as the frontline diagnostic modality in primary care, remote regions, and intraoperative settings worldwide. To address this diagnostic disparity, we developed a deep learning algorithm optimized for rapid fracture screening in resource-limited environments—where 92% of initial fracture presentations occur in developing nations (China National Health Commission, 2023). Our approach leverages an important epidemiological reality: while X-ray equipment achieves universal penetration in China’s primary care facilities, CT availability remains constrained to 58% of these settings. Through a validated radiographic classification system—with all interpretations confirmed by both CT and senior orthopedic specialists (k = 0.86, 95%CI 0.83–0.89)—we demonstrate diagnostic consistency comparable to gold-standard CT (89.7% agreement). The clinical impact is substantial: our method reduces time-to-diagnosis by 72% (Δ = 42.5 ± 3.2 min; *P* < 0.001 by Wilcoxon signed-rank test) while maintaining 94.3% accuracy for non-complex fractures relative to CT. These advances hold particular promise for emergency triage systems and medical training programs in underserved regions. Future directions should prioritize: creation of multimodal imaging repositories, refinement of surgical planning annotations, and multinational validation trials to establish generalizability across diverse healthcare ecosystems. While the SL model demonstrated accuracy comparable to senior clinicians within the retrospective dataset, it is important to acknowledge that retrospective data may differ from real-time clinical scenarios in several ways. Therefore, the model’s performance might differ in different prospective study, as it would be subject to these variables, which are typically not present in retrospective datasets.

Although there is currently a lot of research on the application of AI in orthopedics, there is a lack of practical integration research between SL and blockchain in the context of data security. This study provides inspiration and potential value for the future direction of trustworthy and secure AI in the medical field. By sharing parameters, this can alleviate the dependence of some smart hospitals on powerful hardware and potentially enable the SL trained model to be applied to remote consultation assistance. This will have a significant impact on enhancing the medical level of doctors and providing high quality telemedicine in developing countries (7, 8, 17, 19–22, 28, 29, 34, 50). From the perspective of technology, the majority of prior studies employed the FL approach for joint learning, utilizing a single agent node to process and update the training parameters of each model. As illustrated in Supplementary Table S3. When conducting local centralized training, the model architectures utilized were Weakly-supervised learning, Semi-supervised learning, and U-net. However, weakly supervised and semi-supervised learning methods depend on distribution assumptions of the data, which may not hold true in real-world scenarios. Moreover, in distributed collaborative training, traditional FL methods for medical image analysis lack robust privacy protection and attack resistance mechanisms, making it challenging to prevent malicious node behavior (29, 33, 34, 40, 57). While they have explored the blockchain-based FL architectures for medical analysis, such as Moulahi et al. (28) using Multi-layer Perceptron for monitoring blood glucose, Kumar et al. (42) employing Swin UNetR for COVID-19 image recognition, and Kumar et al. (50) applying U-net for brain tumor image segmentation, these models still face limitations in smart contract programing that restricts flexible data authorization. Recent studies have adopted the SL model to converge dynamic parameters (24–26), with distributed uploads of model update parameters through multiple nodes. Nevertheless, from the perspective of data acquisition, most of these studies only rely on repeated sampling from public databases, such as TCGA, BraTS 2017, and GEO database, resulting in trained models lacking real-world clinical validation (23–26, 29, 33, 34, 39, 44, 45, 55). In the medical field, obtaining labeled data is both costly and requires professional expertise. To address this challenge, we recruited orthopedic surgeons and imaging physicians from multi-center hospitals in China to collaboratively annotate datasets, establishing the first disease-specific database for bone fracture detection.

Current research on polycentric knowledge graphs predominantly focuses on joint embedding learning, which trains embedded models without centralizing various knowledge graphs to ensure data security (6, 8, 13, 53, 56). In this study, we propose a knowledge graph system framework based on the YOLO algorithm designed to promote collaboration among multiple centers without sharing raw data, thereby enabling a comprehensive assessment of fracture patients. First, our approach emphasizes the collaboration of local models rather than the sharing of original images. By contrast, existing research has primarily concentrated on securely sharing model parameters through blockchain and selective encryption, which often faces challenges related to data privacy and intellectual property rights. Second, our proposed framework utilizes multicenter imaging data at the application stage, as opposed to relying on public databases, making it more closely aligned with clinical reality. Models derived from public databases frequently fail to accurately analyze real-world cases when applied in clinical practice. We demonstrated the feasibility of applying a distributed orthopedic diagnostic model in real clinical settings. Third, our proposed approach employs the SL model to summarize local model parameters and reason about local clinical findings. The proposed method addresses existing data gaps, ensures data privacy and security, and provides robust anti-attack capabilities. To the best of our knowledge, no studies have addressed the distributed AI model training and collaboration of medical images during clinical decision support in orthopedic emergency settings (24–26, 58–61). We introduced a pilot framework and reported clinical application results demonstrating the value of using multicenter image data for fracture evaluation in dencentralized way. This approach may assist orthopedic surgeons on the front lines of emergency care and in remote regions in enhancing both efficiency and diagnostic accuracy, potentially allocating more valuable time for the treatment of trauma patients, thereby enhancing the effectiveness of medical interventions.

## 6 Limitations and future work

While this study demonstrates the potential of decentralized learning for fracture classification, several limitations must be acknowledged: the retrospective design may introduce confounding factors, manual image annotation carries inherent subjectivity, data imbalance across nodes presents classification challenges, particularly for rare fracture subtypes like unclassified traumatic TPF, and the current framework lacks systematic incentive mechanisms for multi-center collaboration – all of which represent important avenues for future research to enhance model robustness and clinical applicability. The integration of blockchain-secured SL into orthopedic diagnostics also encounters regulatory considerations, these include computational bottlenecks in real-time segmentation of complex fracture patterns during cross-institutional model synchronization; irreconcilable tensions between ensuring radiological data immutability and adhering to musculoskeletal imaging privacy protocols under the Health Insurance Portability and Accountability Act (HIPAA) Security Rule and General Data Protection Regulation (GDPR). Addressing these challenges requires both technical advancements in Byzantine fault-tolerant consensus mechanisms and prospective validation through international orthopedic trauma registries to establish clinical feasibility.

Regarding the scalability of the model, it is essential to further amass more scarce medical records and image data based on the SL model for exploring multi-site and multi-type fractures, such as the intricate classification of spinal and pelvic fractures, as well as the early prediction of latent fractures and bone metastatic tumor fractures. Integrating SL servers into the existing infrastructure in diverse institutions of multiple countries might entail considerable practical efforts, which need to be addressed through the collaboration of nodes in the consortium chain. To evaluate the compatibility of the SL collaborative network with data and its willingness to be applied in practical scenarios, it is necessary to validate this technology on a larger scale among international societies, hospitals, and organizations. Furthermore, exploring incentive mechanisms for institutional collaboration is crucial. Full stakeholder participation is necessary to encourage the adoption of this innovative architecture, enabling the creation of a trusted, distributed model. When deploying the system across multiple hospitals, challenges in communication efficiency and potential bottlenecks may arise. Additionally, as the system scales, the costs related to network and computational resources may increase, particularly due to the alignment of semantic reasoning across multiple images. To address these challenges, further refinement of the Hyperledger framework could support the broader deployment of the system.

## 7 Conclusion

This study establish SL as a robust framework for privacy-preserving decentralized AI in medical imaging, demonstrating its clinical utility through optimized deep learning nodes. We achieve precise visual localization of fracture patterns with surgical-level accuracy. Automated diagnostic support may significantly reduce the workload of radiologists, while securely enabling multi-institutional data collaboration without compromising patient confidentiality. Systematic validation against state-of-the-art solutions reveals superior performance in diagnostic accuracy, computational efficiency, and clinical workflow integration—particularly for osteosurgical cases requiring preoperative planning. By overcoming traditional barriers to data sharing while maintaining the medical data compliance, our SL paradigm provides a scalable solution for global fracture diagnostics, offering both technical and practical advancements over existing FL approaches. These findings position decentralized AI as a transformative tool for orthopedic imaging, with applications in trauma centers and potential extensions to other image-guided surgical specialties.

## Data Availability

The original contributions presented in the study are included in the article/Supplementary material, further inquiries can be directed to the corresponding authors.

## Footnotes

1
https://github.com/jaysontree/fedv_learning
