# Supplementary material for: Swarm learning network for privacy-preserving and collaborative deep learning assisted diagnosis of fracture: a multi-center diagnostic study
Source: Front Med (Lausanne). 2025 Jul 3;12:1534117. doi: 10.3389/fmed.2025.1534117 (PMC12267170; doi:10.3389/fmed.2025.1534117)
Supplement: Supplementary file 1 [file Data_Sheet_1.docx]

**Table S1**. The swarm learning characteristics over conventional AI and FL [15,16,19,20,22-28].

|  | **AI** | **FL** | **SL** |
| --- | --- | --- | --- |
| **Decentralization** | Relies on centralized big data for deep learning modeling. It is difficult in the collection and organization of data, even has the risk of data leakage and legal liability. | Rely on centralized servers and vulnerable to compromise by malicious users, may be insecure. | Store model updates in a resistant and fault-tolerant manner, resilient against single point of failure attacks. |
| **Traceability** | Only responsible for extracting data features. It cannot distinguish the authenticity and source of the data, requires manual screening and verification. | Only store the most recent model, impossible to hold participants accountable or conduct audits. | Keeps the history of all blocks linked into a chain, participants cannot deny the authorship of model updates. |
| **Immutability** | The medical data can be tampered with and easily falsified; difficult to realize continuous accounting and filing. | Historical model training updates can be tampered with by a malicious server, and hard to detect. | The tempering of records in SL is detectable and blocked by the server. Each block contains a unique hash value to ensure its permanence and unalterability. |
| **Incentives** | Non-incentive mechanism. It can’t benefits participants in the annotation and data contribution. | There is no specific incentive mechanism to encourage all parties to participate in the relevant model training. | Rewards or incentives mechanisms, participants are attracted and contributed with quality data model updates, resulting in an accurate global model. |
| **Integrity and reliability** | Difficult to guarantee the authenticity and transparency of medical data sources. The applicability and credibility of the model cannot be further improved. | Training tasks are coordinated by a single central server, and the results are susceptible to malicious actors or certain servers. | All blocks are connected cryptographically, in case of data alteration they can be detected easily. Blockchain proves as an inherently secure and reliable technology. |
| **Trust** | Centralized model training, individual medical institutions can not monitor each other and dynamic summary. | Do not provide any consensus algorithms or protocols for training design models. | Using consensus algorithms to build trust between parties, participants can participate in different rounds of training. |

**Table S2.** The included and excluded content.

| **Inclusion criteria** | **Exclusion criteria** |
| --- | --- |
| 1.Patients were adults (age >18 years old) | Juvenile patients (age <18 years old) |
| 2.No other knee fractures and chronic pain were associated (such as the fractures of the distal femoral fracture,osteoarthritis, and osteomyelitis involves the tibial plateau) | Combined with other diseases affecting the diagnosis of fracture were associated |
| 3.The preoperative anteroposterior X-ray was available and standard without any improper position, overexposure, ghosting, and shelters, such as plaster, splint, and metal objects on clothes | The preoperative anteroposterior X-ray was not performed in the hospital or no standard |

**Table S3**. Summary of fracture detection performance of different versions of YOLOv8 model at optimal thresholds.

|  | **Optimal thresholds** | **Accuracy (95% CI)** | **Sensitivity (95% CI)** | **Specificity (95% CI)** | **Youden index (95% CI)** |
| --- | --- | --- | --- | --- | --- |
| YOLOv8-n | 0.5493 | 0.9632 (0.9469, 0.9815) | 0.9884 (0.9769, 0.9968) | 0.9366 (0.9242, 0.9468) | 0.9290 (0.9054, 0.9427) |
| YOLOv8-s | 0.6042 | 0.9630 (0.9526, 0.9725) | 0.9603 (0.9431, 0.9815) | 0.9610 (0.9366, 0.9865) | 0.9263 (0.9054, 0.9481) |
| YOLOv8-m | 0.5646 | 0.9550 (0.9388, 0.9730) | 0.9711 (0.9513, 0.9846) | 0.9415 (0.9691, 0.9967) | 0.9126 (0.8889, 0.9335) |
| YOLOv8-l | 0.6033 | 0.9524 (0.9360, 0.9757) | 0.9538 (0.9123, 0.9673) | 0.9512 (0.9613, 0.9932) | 0.9050 (0.8806, 0.9289) |
| YOLOv8-x | 0.5722 | 0.9630 (0.9361, 0.9822) | 0.9769 (0.9488, 0.9886) | 0.9532 (0.9309, 0.9770) | 0.9281 (0.8884, 0.9544) |

**Table S4**. Summary of the performance of different models in the classification of TPF.

|  | **Average**  **accuracy** | **Accuracy in classification** | | | | |
| --- | --- | --- | --- | --- | --- | --- |
|  |  | **Type A** | **Type B** | **Type C** | **Type D** | **Type K** |
| **YOLOv8n-cls** | 0.8441 | 0.8360  (0.8198, 0.8413) | 0.8221  (0.8169, 0.8418) | 0.8725  (0.8469, 0.8815) | 0.8553  (0.8429, 0.8726) | 0.9220  (0.9061, 0.9414) |
| **YOLOv8s-cls** | 0.7516 | 0.7112  (0.6969, 0.7218) | 0.7903  (0.7699, 0.8103) | 0.7005  (0.6897, 0.7125) | 0.7786  (0.7655, 0.7818) | 0.6755  (0.6654, 0.6868) |
| **YOLOv8m-cls** | 0.7514 | 0.6771  (0.6517, 0.6824) | 0.7918  (0.7803, 0.8029) | 0.7021  (0.6801, 0.7125) | 0.7774  (0.7589, 0.7821) | 0.7556  (0.7412, 0.7751) |
| **YOLOv8l-cls** | 0.7572 | 0.6772  (0.6521, 0.6885) | 0.8006  (0.7869, 0.8215) | 0.7056  (0.6969, 0.7221) | 0.7786  (0.7654, 0.7863) | 0.7560  (0.7433, 0.7642) |
| **YOLOv8x-cls** | 0.7571 | 0.7115  (0.7012, 0.7344) | 0.7740  (0.7566, 0.7825) | 0.7467  (0.7212, 0.7599) | 0.8121  (0.7769, 0.8256) | 0.5887  (0.5633, 0.6235) |
| **Densenet-161** | 0.7460 | 0.7131  (0.6895, 0.7202) | 0.7903  (0.7801, 0.8058) | 0.7458  (0.7262, 0.7568) | 0.7720  (0.7563, 0.7897) | 0.5043  (0.4898, 0.5188) |
| **ResNet-50** | 0.6886 | 0.5865  (0.5625, 0.6077) | 0.7212  (0.6856, 0.7488) | 0.7067  (0.6912, 0.7256) | 0.7565  (0.7322, 0.7816) | 0.4232  (0.4219, 0.4403) |
| **Swin-s** | 0.7405 | 0.6732  (0.6642, 0.6858) | 0.7767  (0.7517, 0.7961) | 0.7458  (0.7215, 0.7644) | 0.7903  (0.7808, 0.7989) | 0.5050  (0.4981, 0.5103) |
| **Efficient-B5** | 0.7058 | 0.6303  (0.6288, 0.6403) | 0.7478  (0.7345, 0.7628) | 0.6519  (0.6389, 0.6725) | 0.7789  (0.7652, 0.7859) | 0.5068  (0.4915, 0.5106) |

**
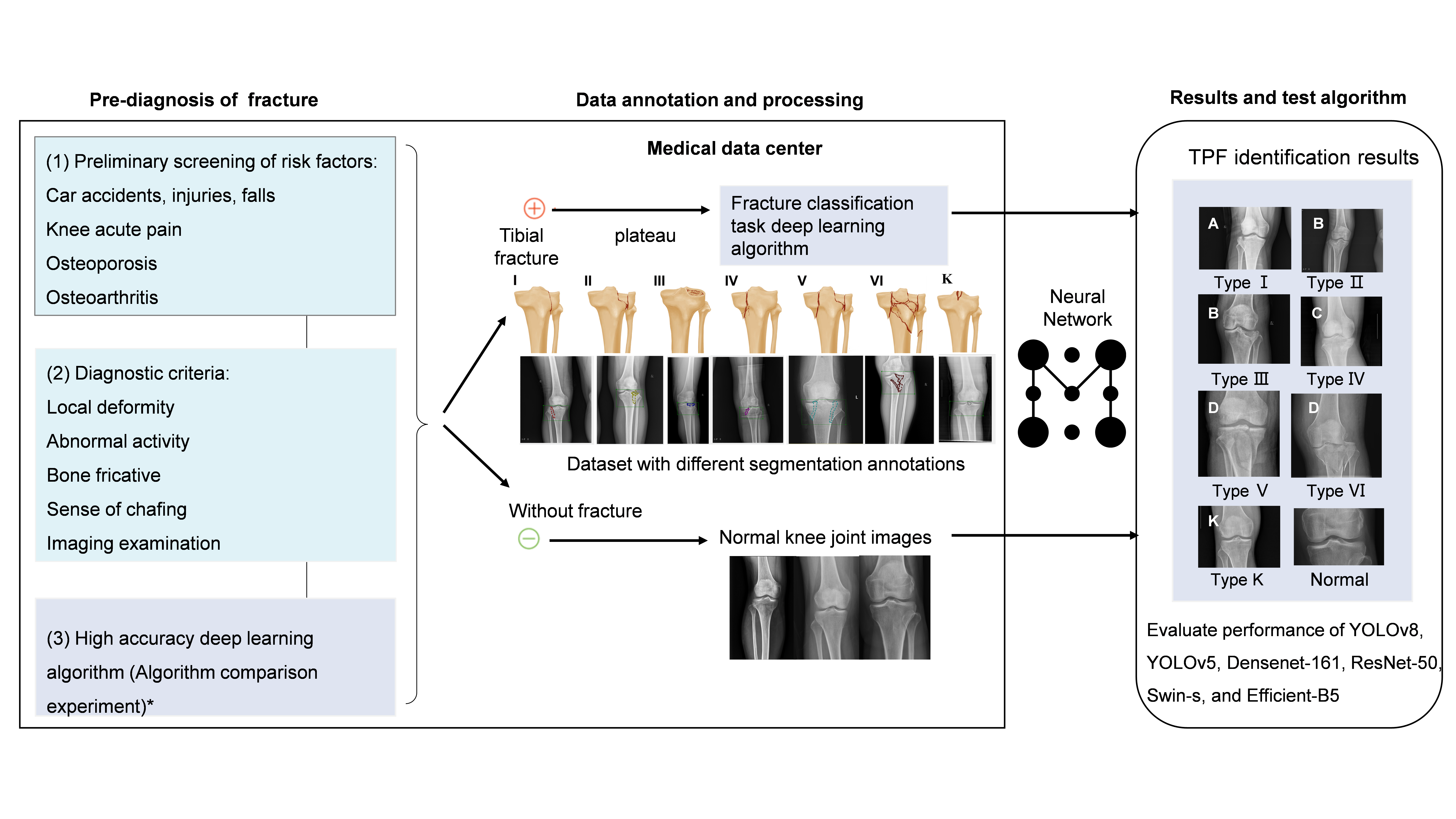
**

**Figure S1** Data preprocessing and identification workflow of normal knee joint and TPF images. The bold white letters ABCDK on the right represent: A, Schatzker Type I, split wedge of the lateral tibial plateau. B, Schatzker Type II, split wedge depression of the lateral tibial plateau; Schatzker Type III, pure depression of the lateral tibial plateau. C, Schatzker Type IV: split wedge of the medial tibial plateau. D, Schatzker Type V: bicondylar tibial plateau fracture, where there is continuity between the epiphysis and the diaphysis; Schatzker Type VI: bicondylar fracture with complete dissociation between the epiphysis and the diaphysis. K, Type K: not in Schatzker Type, intercondylar crest fracture of the tibial plateau. TPF, tibial plateau fracture; SL, swarm learning; DL, deep learning.


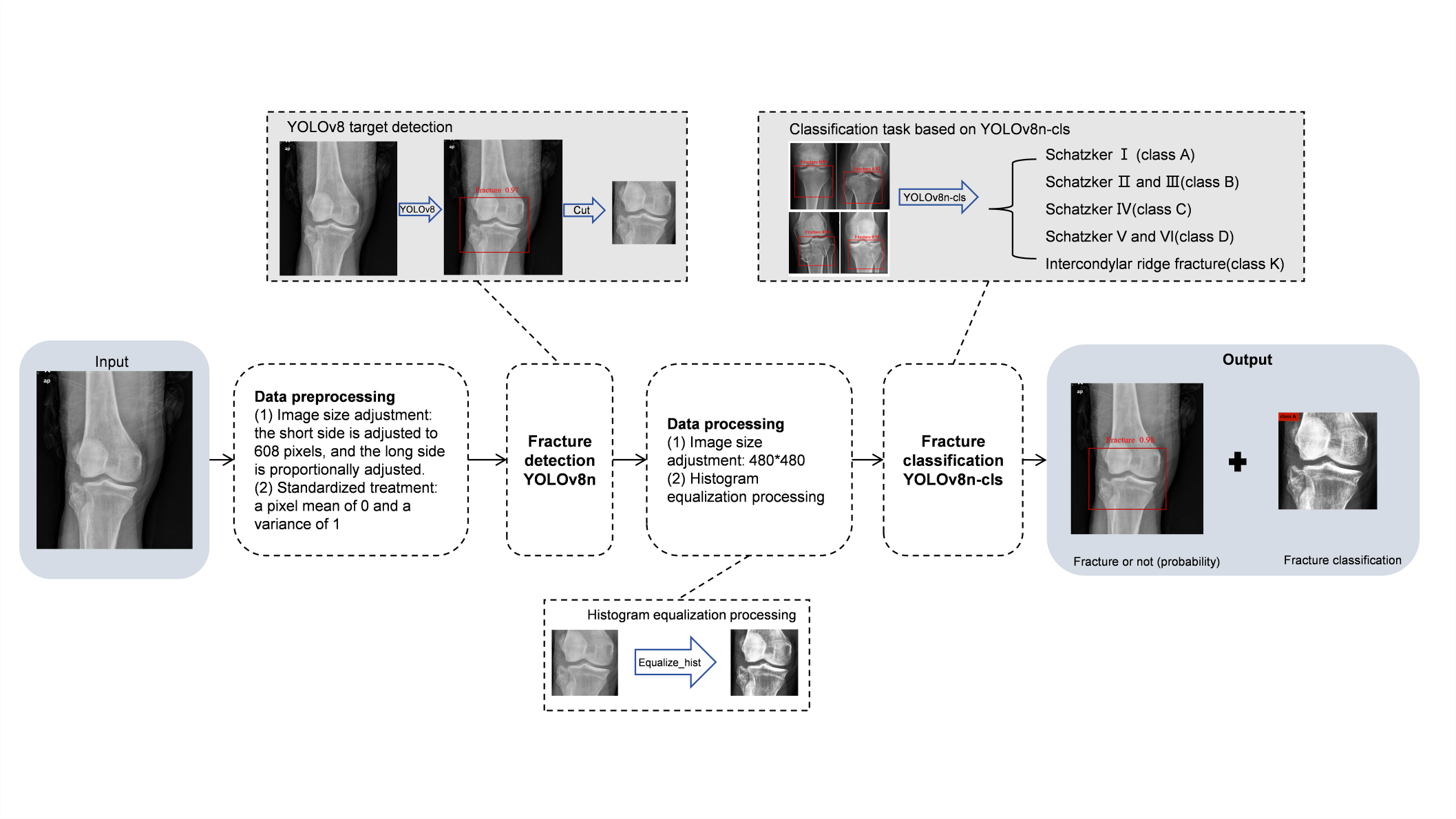


**Figure S2** TPF detection model development and evaluation process.

**
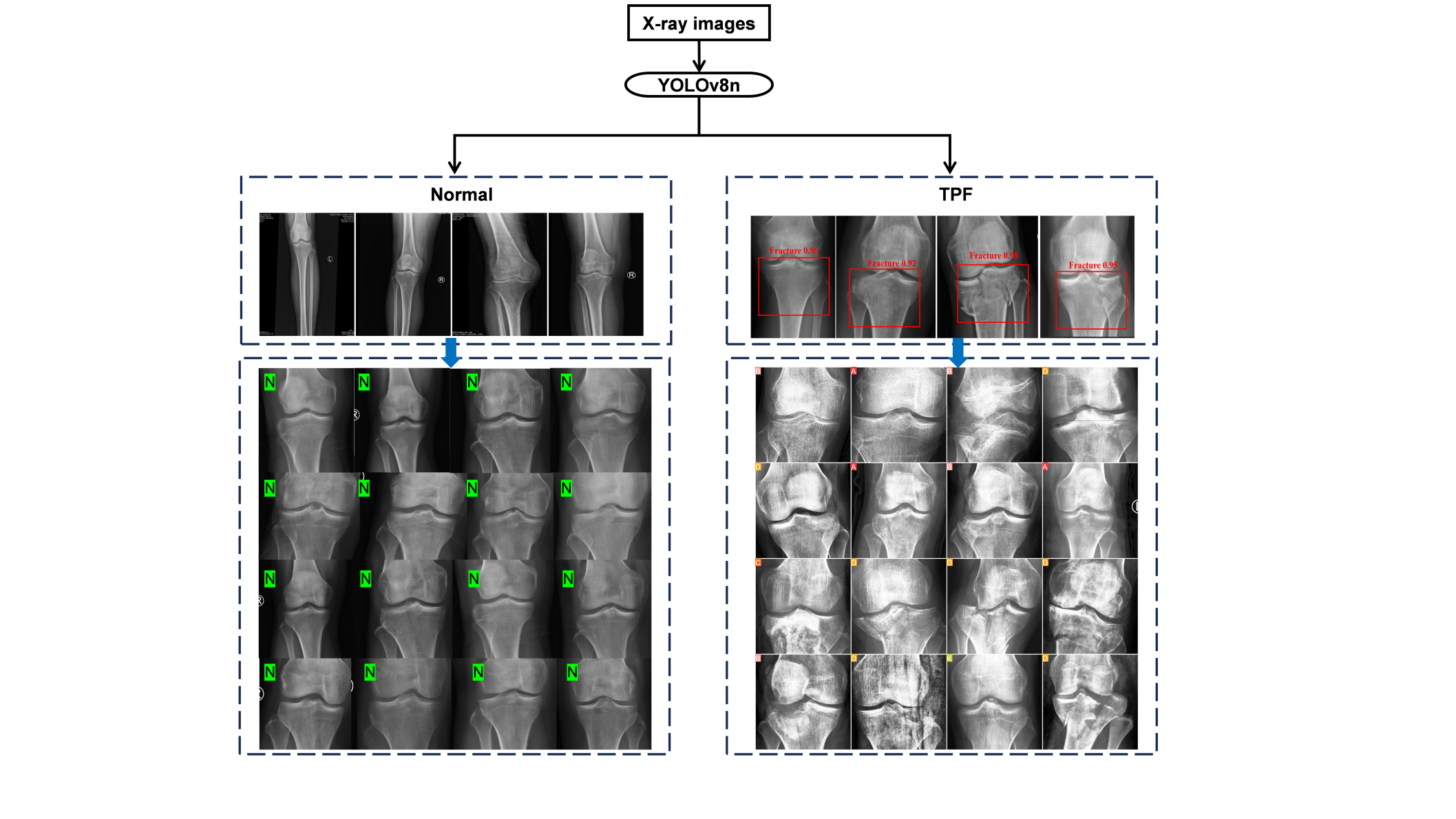
**

**Figure S3** Partial visualization results of fracture detection and classification.
